# Solar-Powered Photodegradation of Pollutant Dyes Using Silver-Embedded Porous TiO_2_ Nanofibers

**DOI:** 10.3390/nano11040856

**Published:** 2021-03-27

**Authors:** Jerry Zhi Xiong Heng, Karen Yuanting Tang, Michelle D. Regulacio, Ming Lin, Xian Jun Loh, Zibiao Li, Enyi Ye

**Affiliations:** 1 Institute of Materials Research and Engineering, Agency for Science, Technology and Research (A*STAR), 2 Fusionopolis Way, Singapore 138634, Singapore; jerry_heng_from.tp@imre.a-star.edu.sg (J.Z.X.H.); tangytk@imre.a-star.edu.sg (K.Y.T.); 2 Institute of Chemistry, University of the Philippines Diliman, Quezon City 1101, Philippines; mdregulacio@up.edu.ph

**Keywords:** photocatalytic oxidation, degradation of organic dye pollutants, localized surface plasmon resonance effect, heterogenous catalysis, hybrid nanocomposites, mesoporous TiO_2_

## Abstract

Titanium dioxide (TiO_2_) nanomaterials have been ubiquitously investigated as a photocatalyst for organic contaminant treatment in wastewater due to their exemplary semiconductor properties. However, their huge band gap remains a barrier for visible light absorption, limiting their utility in practical applications. The incorporation of noble metals in the TiO_2_ scaffold would help mitigate the problem via plasmonic resonance enhancements. Silver (Ag) is the chosen noble metal as it is relatively cheap and has great plasmonic effects. In this study, the use of electrospun Ag-embedded TiO_2_ nanofibers as a photocatalyst is shown to be effective in decomposing rhodamine B and methyl orange dyes under a solar simulator in 3 h, which is more efficacious as opposed to pristine TiO_2_ nanofibers. This showcases the potential of a simple and economic wastewater treatment system for the removal of organic pollutants.

## 1. Introduction

Organic dyes have a significant foothold in our everyday lives, as they provide vibrant colors to a range of commodities, from textiles to cosmetics to pharmaceuticals [1]. In addition, they are employed in the forefront of photovoltaic [2] and optics research [3,4]. However, the ubiquitous use of these organic dyes also makes them a major component in industrial effluents, causing widespread wastewater contamination. A few case studies of dye concentrations in textile wastewater were performed, with typical dye concentrations reported in ranges between 10 and 250 mg/L [5,6]. These organic compounds are highly soluble in water and non-biodegradable, remaining in water bodies for extensive periods of time. This could lead to bioaccumulation in aquatic life and biomagnification along the food chain [7]. Additionally, these compounds typically absorb in the visible light region, permitting less light transmission and hindering the photosynthesis of aquatic flora. Hence, it is imperative that accessible technologies are innovated to resolve the contemporary problem of organic dye contamination in wastewater. 

Recently, the employment of metal oxide semiconductors, such as TiO_2_ and ZnO, has been touted as an efficacious and environmentally friendly approach to treating organic matter such as dye pollutants present in wastewater [8]. By harnessing light, they are able to degrade the dye pollutants at ambient temperatures and pressure [9]. Its methodology involves the formation of highly reactive hydroxyl radicals from the absorption of photons through electron–hole pairs, which are imperative in oxidizing the organic dyes into milder and less hazardous substances. In this aspect, nanocomposites of TiO_2_ have been vastly utilized as they entail various favorable properties, such as reusability, inertness, and economic and ease of accessibility [10]. Previous studies have shown the heterogeneous catalysts’ capability in decomposing a handful of common dyes, such as methylene blue (MB), methyl orange (MO), and azo dyes under ultraviolet (UV) radiation [11,12]. While respectable, its functionality and potential for industrialization could be vastly improved with an additional ability to tap on visible light for generating charge carriers. This allows the exploitation of sunlight, a readily available light source, which is composed of both UV and visible light radiation. 

The huge band gap of TiO_2_ (E_g_ = 3.2 eV for anatase and 3.0 eV for rutile) restricts its absorption of electromagnetic radiation only to the UV region. Doping of the semiconductor could narrow the band gap for visible light absorption [13]. Alternatively, the presence of complementary noble metal nanoparticles (NPs) could supplement the semiconductor in absorbing visible light atop of strengthened UV radiation absorption. For this purpose, hybrid nanocomposites comprising both adducts have been engineered [14], where the noble metal NPs could benefit the TiO_2_ matrix two-fold. Firstly, the hybrid nanocomposite can synergistically tap on both UV and visible light as the localized surface plasmon resonance (LSPR) phenomenon enables noble metal NPs to intensely absorb in the visible region. This is due to the small particle effect [15] and is brought about by the synchronized oscillation of electrons induced by incident photons at resonant frequency [16,17,18,19,20,21]. This is followed by the insertion of highly energized electrons in the conduction band of TiO_2_, which increases the charge carrier density and enhances photocatalytic activity. Secondly, as the conduction band of TiO_2_ occurs at a higher energy than the Fermi energy of noble metal NPs, the noble metal NPs can moderate recombination rates in TiO_2_ by facilitating a charge transfer process at the heterojunction of the Ag-TiO_2_ hybrid, since they could serve as a sink for charge carriers that was generated by the absorption of light [22]. This allows more electron–hole pairs to be maintained, which again enhances photocatalytic activity. In brief, the presence of a noble metal NP component mitigates the TiO_2_ semiconductor’s drawbacks and supplements it to become a more efficient photocatalyst.

Among the various metals [20,23,24,25,26], Ag NPs are a suitable candidate to be incorporated into the TiO_2_ matrix for photocatalytic applications. Besides being cheap and relatively abundant, they boast an intense SPR effect that can offer superior plasmonic enhancement to the TiO_2_ photocatalyst. The combination has been subjected to a couple of dye degradation studies for this reason [27,28,29]. Meanwhile, one-dimensional (1D) nanofibers serve as an appealing architecture for this application, as they can be convenient for separation and recovery [30,31]. Through the electrospinning method, nanofibers with a uniform deposition of Ag NPs can be fabricated inexpensively and even on an industrial scale. In this method, a syringe pump is used to spin a solution containing component adducts into an extrusion of nanofibers, assisted by an electric current [32]. Thus, the pairing of Ag NPs with TiO_2_ nanofibers would be pertinent in the development of a low-cost, accessible, and scalable solution to dye pollutant degradation.

Despite numerous precedents reporting on dye pollutant decomposition with TiO_2_ nanocomposites, the utilization of Ag NPs-embedded TiO_2_ nanofibers is scarce for this purpose [33,34,35]. Hence, these nanofibers were assessed in this study for their ability to harness solar energy and degrade two representative organic dyes, namely RhB and MO, providing us with valuable insights on the photocatalyst’s performance on xanthene and azo classes of dye, respectively. Most importantly, we strive to integrate the use of cheap, safe, and accessible materials with facile methodologies to combat dye pollutant contamination in wastewater.

## 2. Materials and Methods

### 2.1. Fabrication of Ag-TiO_2_ Nanofibers

The Ag-TiO_2_ nanofibers were fabricated in accordance with our previous work [32]. Typically, 0.32 mL of titanium tetraisopropoxide (TTIP) was added with 0.6 mL of ethanol and 0.6 mL of acetic acid in a glovebox. The pale yellow solution was then added to another mixture consisting of 0.18 g of polyvinylpyrrolidone (PVP, MW = 1,300,000), 1.5 mL of ethanol, and 17.0 mg of AgNO_3_. The resultant mixture was vigorously stirred for 1 h to afford a homogeneous solution, readied for electrospinning. After loading the solution into a 6 mL syringe with a blunt 22-gauge needle (inner diameter of 0.413 mm), it was spun at a constant rate of 0.8 mL/h with a voltage of 12 kV applied to the needle, producing nanofibers on an aluminum foil (collector) with a distance of 20 cm away from the needle. The nanofibers were left in ambient conditions overnight to allow the full hydrolysis of TTIP and were subsequently calcined at 500 °C in air for 3 h to remove PVP and other carbonaceous materials. After calcination, the color of the nanofibers turned from white to bluish violet. In the absence of AgNO_3_, the protocol was replicated for pristine TiO_2_, which acts as the control for the experiment, affording white nanofibers after calcination.

### 2.2. Characterization of Pristine TiO_2_ and Ag-TiO_2_ Nanofibers

In obtaining the X-ray diffraction patterns for both the Ag-TiO_2_ and pristine TiO_2_ nanofibers, they were placed on a 1 × 1 cm^2^ silicon wafer and measured using a Bruker GADDS D8 Discover diffractometer (Bruker Corp., Billerica, MA, USA). Cu_Ka_ radiation was used at a working voltage and current of 40 kV and 40 mA, respectively. Next, the morphology of the nanofibers was characterized using a JEOL JSM 6700F field emission scanning electron microscope (FESEM; JEOL Ltd., Tokyo, Japan) in transmission mode. The samples were coated with a thin layer of Au via sputtering prior to imaging. The morphology of the nanofibers was additionally analyzed using a FEI Titan transmission electron microscope (TEM; Thermo Fischer Scientific, Waltham, MA, USA). Bright-field TEM images were collected at an accelerating voltage of 200 kV. High-angle annular dark-field scanning TEM (HAADF-STEM) images of both nanofibers were also acquired using the STEM mode. For the UV-Vis absorption measurements, the nanofibers were dispersed in deionized water under sonication for 2 min prior to analysis. The absorption spectra were recorded at ambient temperature using a Shimadzu UV-1800 spectrophotometer (Shimadzu Coroporation, Kyoto, Japan).

### 2.3. Photocatalytic Degradation Studies

The photocatalytic performance of the Ag-TiO_2_ nanofibers was evaluated in the photocatalytic degradation of rhodamine B (RhB, Sigma-Aldrich, ≥95%) and methyl orange (MO, Sigma-Aldrich, ≥85%). In a typical experiment, 5 mg of the nanofibers was dispersed in a 50 mL aqueous solution of the dye (RhB or MO), and the resulting mixture was left to stir for 1 h in the dark to establish an adsorption–desorption equilibrium. The mixture was then irradiated using a solar simulator (AM 1.5G). At specified time intervals, a 1 mL aliquot of the mixture was periodically taken out and then centrifuged to remove the nanofiber photocatalyst. The absorption spectra of the supernatant were measured using a Shimadzu UV-1800 spectrophotometer to monitor the photocatalytic degradation process. Prior to each experiment, a blank sample of the dye was taken for calibration purposes. The change in concentrations for the dyes RhB and MO was monitored by measuring the absorbance at 553 nm and 463 nm, respectively. The extent of degradation over time was determined by calculating C/C_0_, where C_0_ and C are the absorbance of the dye solution before and after irradiation, respectively. For comparison purposes, the photocatalytic performance of the pristine TiO_2_ nanofibers was also measured under the same experimental conditions.

## 3. Results and Discussion

### 3.1. Synthesis and Characterization of Ag-TiO_2_ Nanofibers

**Figure 1:**
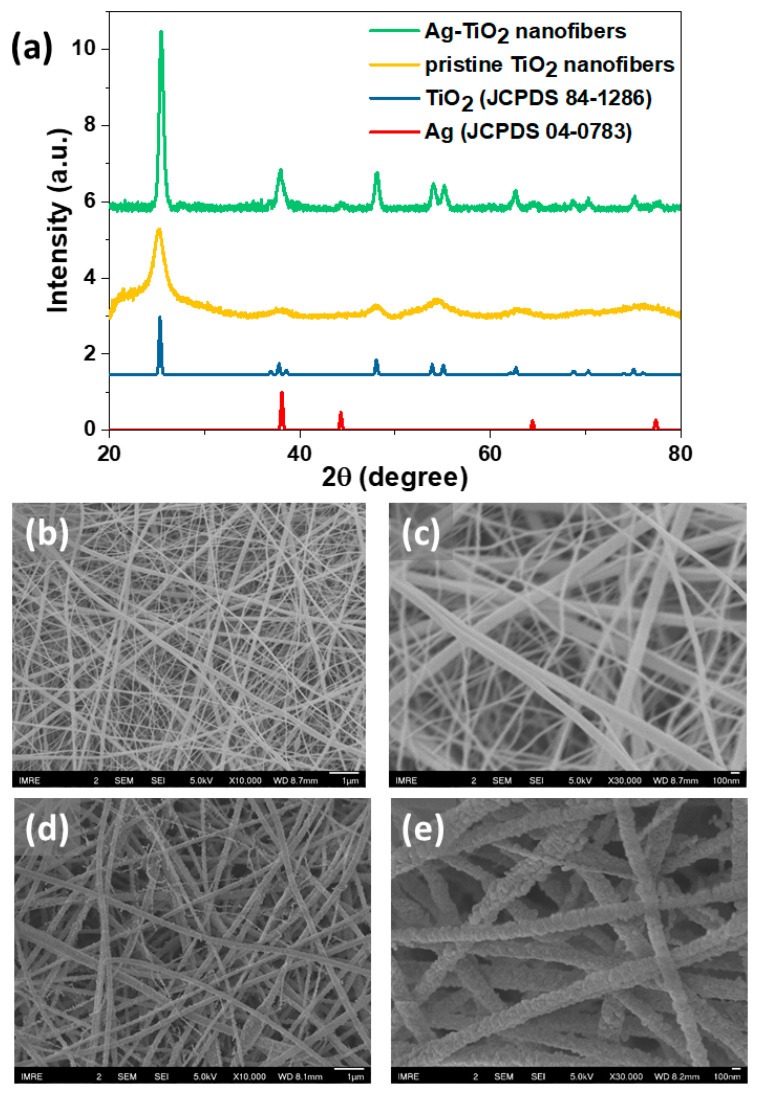
(**a**) XRD patterns for experimental Ag-TiO_2_ nanofibers (green) and pristine TiO_2_ nanofibers (yellow) alongside literature patterns for TiO_2_ (blue) and Ag (red); (**b,c**) SEM images of the pristine TiO_2_ nanofibers after calcination at (**b**) 10,000× and (**c**) 30,000× resolution; (**d,e**) SEM images of the Ag-TiO_2_ nanofibers after calcination at (d) 10,000× and (e) 30,000× resolution.

The Ag-TiO_2_ nanofibers were proficiently fabricated in accordance with a previously reported protocol [32]. With the aid of PVP, TiO_2_ could be spun into a thread-like morphology from a mixture composing of both TiO_2_ and Ag precursors using the electrospinning method. This was followed by the hydrolysis of TTIP upon setting and calcination of the nanofibers to remove PVP and other carbonaceous materials. Figure 1a presents the XRD patterns for experimental Ag-TiO_2_ and pristine TiO_2_ nanofibers after calcination, alongside the patterns in the literature for tetragonal anatase TiO_2_ and face-centered cubic Ag. By comparing the experimental patterns to those in the literature, it could be ascertained that the pristine TiO_2_ nanofibers only contain peaks that coincide with the patterns in the literature for TiO_2_, while additional peaks corresponding to Ag were present for the Ag-TiO_2_ sample. This indicates that the Ag NPs were present within the Ag-TiO_2_ sample. Moreover, the XRD patterns for the two nanofiber samples could be used to deduce the average domain size of the crystals in the samples [36]. For instance, using the Scherrer equation, the full width half maximum (FWHM) of the TiO_2_ signals in the Ag-TiO_2_ sample is approximately twice of that in the pristine TiO_2_ sample. This translates the sharper and more well-defined signals in the Ag-TiO_2_ sample to TiO_2_ crystals being larger and of great crystallinity, in contrast with smaller and more amorphous crystallites in the pristine TiO_2_ sample. Hence, the embedment of Ag NPs potentially aided TiO_2_’s crystallization process.

**Figure 2:**
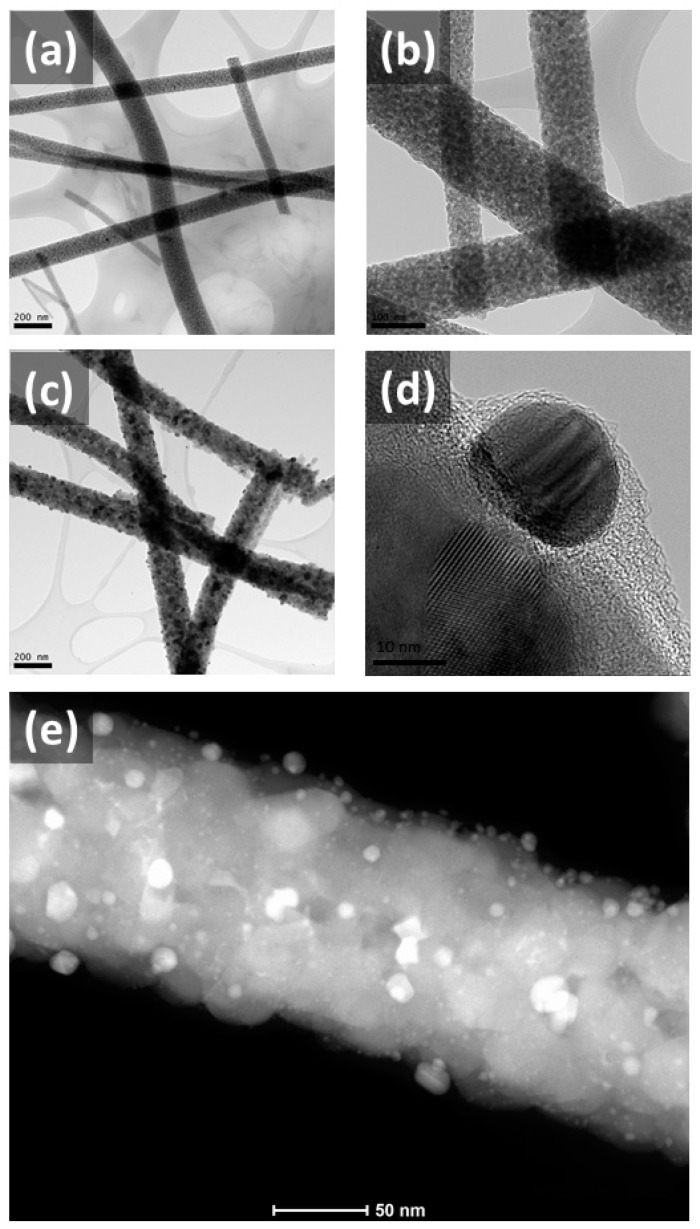
TEM images of (**a,b**) pristine TiO_2_ nanofibers and (**c,d**) Ag-TiO_2_ nanofibers; (**e**) the high-angle annular dark-field scanning TEM (HAADF-STEM) image of the Ag-TiO_2_ nanofibers before the photodegradation study.

The morphologies of pristine TiO_2_ and Ag-TiO_2_ nanofibers were analyzed using SEM and TEM, as illustrated in Figure 1 and Figure 2. It was observed in the SEM images (Figure 1b,c) that both samples displayed a thread-like structure even after calcination, retaining their 1D morphology. From the SEM images, it could be approximated that the average diameter for the pristine TiO_2_ nanofibers and the Ag-TiO_2_ nanofibers is 114 nm and 171 nm, respectively. On closer examination, innumerable protrusions were observed along the surface of Ag-TiO_2_ nanofibers (Figure 1e), resembling “beaded strings”, in contrast to the seemingly even threads in pristine TiO_2_ (Figure 1d). This suggests that the identity of the protrusions is probably be the Ag NPs.

Next, TEM imaging was used to investigate the internal construct of the nanofibers. In Figure 2a,b, a copious number of minute TiO_2_ crystals were randomly located throughout the nanofibers, translating to a ragged surface and mesoporous nature of the photocatalyst. Likewise, this is also shown in the TEM images of Ag-TiO_2_ nanofibers in Figure 2c,d. At greater magnification, spheroidal Ag crystallites of diameters averaging at about 20 nm were observed to randomly agglomerate within the nanofiber scaffold. In addition, high-angle annular dark-field scanning TEM (HAADF-STEM) was performed to further substantiate the findings presented above and to differentiate between Ag and TiO_2_ crystals. As Ag has a greater atomic mass than Ti and O, they turned out to be brighter than the latter. It was thus observed that there was a good distribution of Ag crystallites along the nanofiber scaffold, with a handful anchored at the rim. These crystals were the product of Ag NPs that were propagated after the calcination process, where Ag^+^ ions were reduced into Ag NPs, and impurities, including carbonaceous materials and remnant PVP, were removed. They were seen to be highly crystalline, which concurs with the XRD data mentioned above. The images also demonstrate the protrusions on the Ag NPs present within the nanofibers.

### 3.2. Photocatalytic Degradation of Dyes with Ag-TiO_2_ Nanofibers

**Figure 3:**
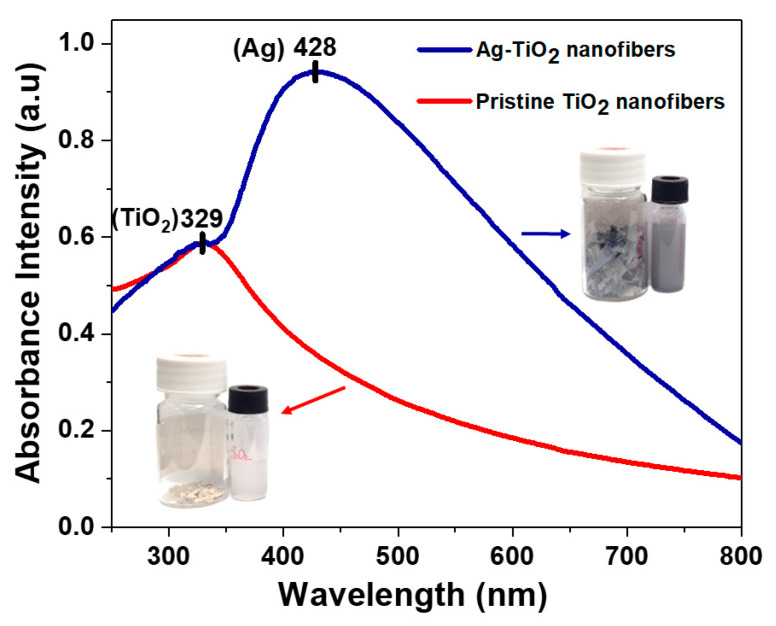
UV-Vis absorption spectra for both Ag-TiO_2_ (blue) and pristine TiO_2_ (red) nanofibers alongside photographs of the nanofibers themselves and when they are dispersed in solvent.

The absorption spectra for both the pristine TiO_2_ and Ag-TiO_2_ nanofibers, dispersed in ethanol, are represented in Figure 3. In both spectra, there is an absorption peak occurring in the UV region at about 329 nm, which corresponds to the absorption by TiO_2_. At longer wavelengths, the pristine TiO_2_ sample saw a decline in absorbance intensity. In contrast, the Ag-TiO_2_ nanofibers absorbed extensively through the visible light region, peaking at 428 nm. Such broadband absorption in the visible region was induced by the LSPR phenomenon and conferred the Ag-TiO_2_ nanofibers its bluish-grey appearance, in contrast to the pristine TiO_2_ nanofibers being white in color. Meanwhile, the absorption maxima at 428 nm of Ag-TiO_2_ implied a red shift in the absorption band since Ag NPs 20 nm in size typically absorb at a wavelength of about 400 nm [37]. This could be attributed to the high refractive index of the TiO_2_ matrix that hosts the immobilized Ag NPs, as the LSPR wavelengths (λ_LSPR_) are influenced by the size of the NPs and the refractive index of the medium [38].

**Figure 4:**
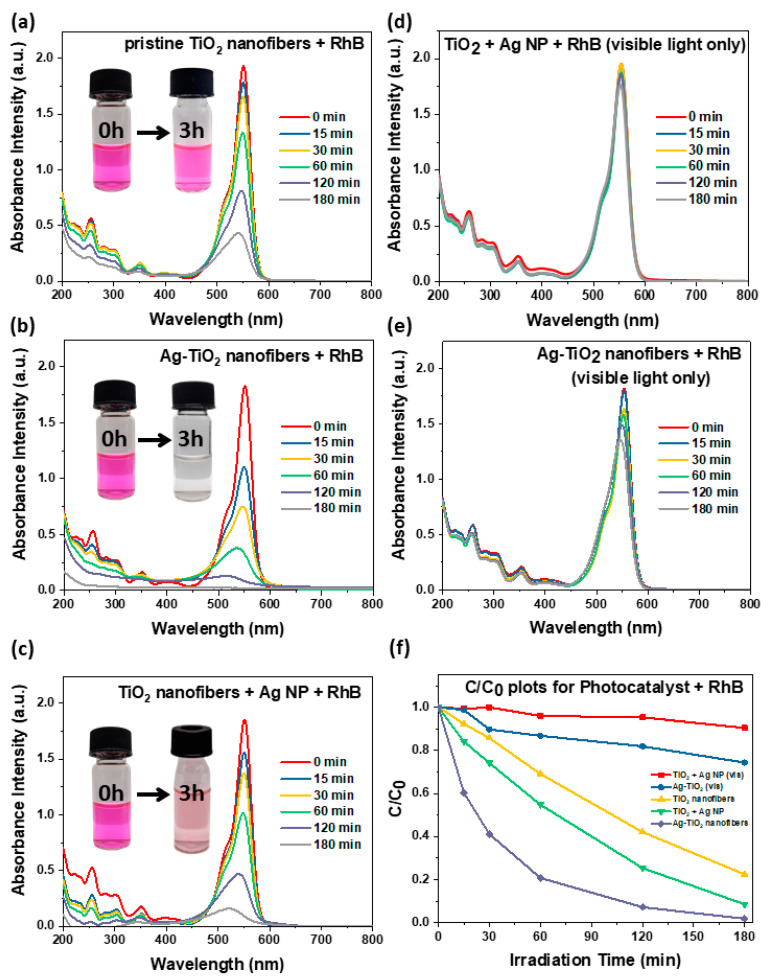
Photodegradation study of rhodamine B (RhB) dye with varying photocatalyst and solar irradiation conditions. For visible-light-only irradiation, a filter was applied to the AM 1.5G solar simulator such that λ_incident_ > 390 nm. (**a**) Use of pristine TiO_2_ nanofibers under solar irradiance; (**b**) Ag-TiO_2_ nanofibers under solar irradiance; (**c**) TiO_2_ nanofibers with extraneously added Ag nanoparticles (NPs) under solar irradiance; (**d**) TiO_2_ nanofibers with extraneously added Ag NPs under visible-light-only irradiance; (**e**) Ag-TiO_2_ nanofibers under visible-light-only irradiance; and (**f**) C/C_0_ plots for the various dye degradation setups with RhB in the following order: TiO_2_ + Ag NPs under visible-only light (red), Ag-TiO_2_ under visible-only light (blue), pristine TiO_2_ nanofibers under full solar irradiation (yellow), TiO_2_ + Ag NPs under full solar irradiation (green), Ag-TiO_2_ nanofibers under full solar irradiation (purple).

After establishing their physical and optical properties, the photocatalytic performance of Ag-TiO_2_ nanofibers was evaluated in a photodegradation study in comparison with pristine TiO_2_ nanofibers. As the photocatalyst and 10 ppm of dye, either RhB or MO, were irradiated under a solar simulator, aliquots of the reaction mixture were sampled periodically, and the results are presented in Figure 4a–c,e–g, respectively. Afterwards, plots of C/C_0_ against irradiation time (Figure 4d,h) were established using the absorbance intensity of the mixture observed at the characteristic absorption peak (λ_max_) for the dyes, 553 nm for RhB and 463 nm for MO, at each sampling interval. By tabulating the C/C_0_ of each sampling interval, we could quantitatively determine the extent of dye that was degraded by the photocatalyst with respect to time. This allowed us to corroborate each photocatalyst’s competence in absorbing incident light and photodegrading the dye substrate compounds.

Figure 4a presents the photocatalytic degradation of RhB dye by pristine TiO_2_ nanofibers, where the absorbance intensity of the RhB absorption peak (536 nm) decreased steadily with solar irradiation. After three hours of irradiation, 77% of the RhB dye in the solution was degraded, and it can be observed in the inset of Figure 4a that the dye turned from an intense shade of pink into a lighter shade. Its C/C_0_ plot in Figure 4f indicates that the photodegradation process proceeded linearly with irradiation time. Next, a similar setup was performed with Ag-TiO_2_ nanofibers (Figure 4b), and it can be seen that as compared to the earlier setup, the RhB dye rapidly decomposed within the first hour (80%), and two subsequent hours of irradiation permitted complete degradation of the dye. This can again be discerned visually in the inset that the dye solution decolorized to a clear solution, indicating that all of the chromophores present had been broken down into simpler products, as elaborated in the later sections.

While the incorporation of Ag NPs within the TiO_2_ matrix yielded positive enhancements to the photocatalyst’s performance, three supplementary experiments were additionally conducted to ascertain the significance of the embedded Ag NPs presented in Figure 4c–e. There are previous reports on the catalytic properties of Ag NPs for the decomposition of harmful dye compounds [39,40]. Thus, in Figure 4c, pristine TiO_2_ was added with synthesized Ag NPs (of similar sizes to that of the embedded Ag NPs in Ag-TiO_2_) and 10 ppm of RhB dye prior to three hours of solar irradiation. It was observed that while there was greater photocatalytic activity seen as compared to pristine TiO_2,_ the setup did not proceed to completion. It could be postulated that the Ag NPs were not in close proximity to the TiO_2_ photocatalyst to provide charge separation/surface plasmon resonance enhancements as optimally as the Ag-TiO_2_ nanofibers. Furthermore, such separation of Ag NPs and TiO_2_ nanofibers complicates recovery of the photocatalyst.

In the next two setups in Figure 4d,e, a filter was applied to the solar simulator to irradiate only visible light on the setups of TiO_2_ + Ag NPs and Ag-TiO_2_ nanofibers, respectively. As expected, there was very little photocatalytic activity observed for the TiO_2_ + Ag NPs sample, as TiO_2_ was not activated with visible light due to its large band gap. Meanwhile, the surface plasmon resonance effect of Ag NPs potentially empowered the Ag-TiO_2_ nanofibers to harness visible light for photocatalysis and exhibit a modest amount of photocatalytic activity. This suggests that the photocatalytic activity in Ag-TiO_2_ nanofibers is predominantly attributed to increased charge separation. More importantly, it concurred that the embedment of Ag NPs within a TiO_2_ matrix is necessary for enhanced photocatalytic activity.

**Figure 5:**
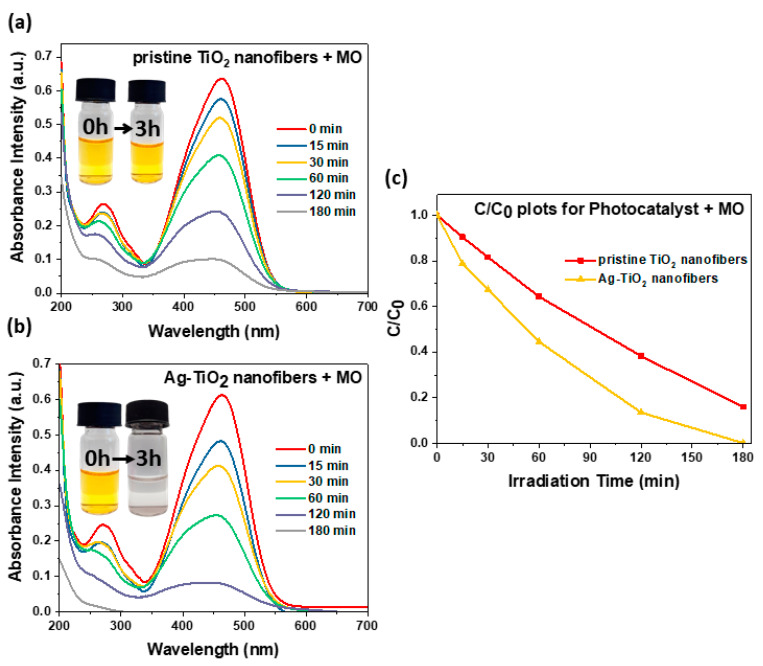
Photodegradation study of methyl orange (MO) dye under solar irradiance with (**a**) pristine TiO_2_ nanofibers and (**b**) Ag-TiO_2_ nanofibers; (**c**) C/C_0_ plots for the two dye degradation setups, where the red line represents the pristine TiO_2_ nanofibers, while the yellow line represents the Ag-TiO_2_ nanofibers.

The photocatalytic study of the three sets of nanofibers was extended to the MO dye (Figure 5), and the outcome was similar, where the C/C_0_ plots for the MO dye mirrored those of the RhB dye. After three hours of irradiation, the MO solution containing the pristine TiO_2_ nanofibers saw an 84% decline (Figure 5a), and complete degradation was again seen with Ag-TiO_2_ (Figure 5b)_._ It is thus evident that incorporation of Ag NPs in TiO_2_ nanofibers simultaneously expedited the photodegradation process and increased its efficacy, presumably brought about by LSPR-induced enhancement and moderation of TiO_2_ recombination rates by the embedded Ag NPs. It is also noteworthy that a gradual blue shift was observed in the dye absorption peaks with irradiation time, attributed to the hypsochromic effect, where removal of conjugation would shift the absorption peak towards shorter wavelengths. The absorption peak for the RhB dye was blue-shifted from 553 nm to 510 nm, containing a mixture of partially de-ethylated intermediates. For example, *N,N′*-diethylated rhodamine and *N-*ethylated rhodamine have absorption peaks at 522 nm and 510 nm, respectively [41]. It could thus be suggested that the final product after the experiment was completely de-ethylated. Similarly, the absorption peak for the MO dye was blue-shifted from 463 nm to 429 nm due to demethylation and cleavage of the azo bond [42].

**Figure 6:**
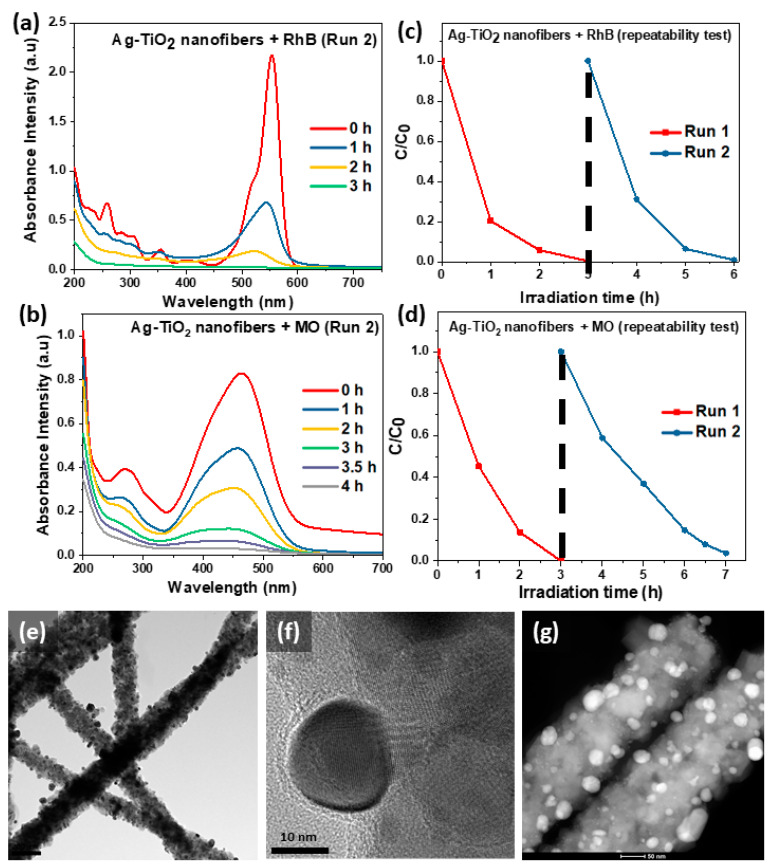
(**a**–**d**) UV-Vis spectra and their respective C/C_0_ against irradiation time plots for the 2nd cycle of photodegradation of RhB and MO dyes with Ag-TiO_2_ nanofibers; (**e,f**) TEM images of the Ag-TiO_2_ nanofibers after the photodegradation study; (**g**) HAADF-STEM image of the Ag-TiO_2_ nanofibers.

We were also interested in exploring the Ag-TiO_2_ nanofibers’ ability to maintain its photocatalytic efficiency when reused. In this, the photocatalyst was recovered from the experiment and subjected to a repeat, as exhibited in Figure 6a–d. Gratifyingly, the Ag-TiO_2_ nanofibers were able to bring about the complete photodegradation of RhB dye and near-complete degradation (99.7%) of MO dye. The TEM images of the photocatalyst after the photodegradation study are also provided in Figure 6e–g, where they are largely identical to the images taken before the experiment. Hence, the results imply that the photocatalyst is robust to be recycled and can be used to service subsequent cycles of dye pollutants photodegradation.

### 3.3. Mechanistic Aspects of the Photocatalytic Degradation Reaction

While Ag-TiO_2_ nanofibers enable dual absorption of UV radiation and visible light from sunlight for enhanced photocatalytic activity, the generation of reactive radical species in each electromagnetic radiation is different [32,42,43]. Both pathways are elaborated in Figure S1. Upon the absorption of UV radiation by TiO_2_ that corresponds to its band gap, excited electrons fill its conduction band (CB), while corresponding holes populate the valence band (VB). Charge separation at the heterojunction of the Ag-TiO_2_ nanocomposite ensues when these electrons are transferred to the embedded Ag NPs. A similar charge separation takes place when highly energetic electrons are inserted into the CB of TiO_2_ from plasmonic Ag NPs after their absorption of visible light. Thereafter, reactive radical species are generated from a series of redox reactions for oxidative degradation of the dye substrates, summarized in Figure S1. The radical chain reaction is initiated when anionic superoxide radicals (·O_2_^−^) are formed from the reduction of dissolved oxygen by free electrons, while hydroxyl radicals (·OH) are formed from oxidation of water by free holes. Through hydroperoxyl radical (HOO·) and hydrogen peroxide (H_2_O_2_) intermediates, O_2_^−^ can be propagated into ·OH radicals. These radicals (·O_2_^−^ and ·OH) are critical in breaking down the dye pollutants into simpler organic molecules, which are exhibited in Figure S2. The organic intermediates for RhB have been identified in previous studies [43]. Further ring opening and mineralization of these intermediates ultimately lead to CO_2_ and H_2_O.

Meanwhile, it has been suggested in recent studies that the surface hydrophilicity of TiO_2_ could facilitate the dye degradation process [44,45]. For instance, Mino and co-workers conducted a FTIR study that investigated the mechanical aspects of phenol degradation on P25 titania [45]. It was discovered in their study that the decomposition of the adsorbed phenol on the titania surface could be influenced by surface hydration conditions and was expedited by one order of magnitude on a hydrated surface. Analogous to phenol, RhB and MO dyes are aromatic compounds with polar substituent groups attached. Hence, the surface hydrophilicity of Ag-TiO_2_ nanofibers could accelerate the dye degradation process.

## 4. Conclusions

In this study, Ag NPs with an average diameter of 20 nm were embedded in TiO_2_ nanofibers, which resulted in successful solar-driven photodegradation of RhB and MO dyes within three hours. The Ag-TiO_2_ nanofibers had showed superior photocatalytic efficiencies when compared to pristine TiO_2_ nanofibers and were aided by two main factors: the Ag NPs’ ability to simultaneously absorb visible light and optimize the charge transfer process in TiO_2_ for improved efficiencies. The photocatalyst could also be conveniently recovered and used for subsequent runs at sustained proficiencies. Besides the degradation of organic dye compounds, the inhibition of microbial growth by the Ag-TiO_2_ nanofibers could be the subject for future studies. Reactive oxygen species, namely ·O_2_ and ·OH, that are generated by Ag-TiO_2_ nanofibers could potentially be antimicrobial agents that could disrupt pathogenic cell functions and cause oxidative damage to the microorganisms. Collectively, this would suggest that the Ag-TiO_2_ nanocomposite may be a simple yet multifunctional solution to the treatment of industrial wastewaters.

## Data Availability

Data is contained within the article and Supplementary Material.

## Supplementary Materials

The following are available online at https://www.mdpi.com/article/10.3390/nano11040856/s1, Figure S1: Schematic overview of the solar powered photocatalytic degradation of RhB and MO dyes with Ag-TiO_2_ nanofibers as the photocatalyst; Figure S2: Proposed photodegradation pathway for RhB and MO dyes by ·OH and ·O_2_^−^ radicals that were generated by electron–hole pairs in Ag-TiO_2_ nanofibers.
