# Supplementary material for: Solar-Powered Photodegradation of Pollutant Dyes Using Silver-Embedded Porous TiO2 Nanofibers
Source: Nanomaterials (Basel). 2021 Mar 27;11(4):856. doi: 10.3390/nano11040856 (PMC8066685; doi:10.3390/nano11040856)
Supplement: Supplementary file 1 [file nanomaterials-11-00856-s001.pdf]

Supplementary Material

Solar-Powered Photodegradation of Pollutant Dyes Using Silver-Embedded Porous TiO_2 Nanofibers

Jerry Zhi Xiong Heng [1,†], Karen Yuanting Tang [1,†], Michelle D. Regulacio [2], Ming Lin [1,*], Xian Jun Loh [1,*], Zibiao Li [1,*] and Enyi Ye [1,*]

1 Institute of Materials Research and Engineering, Agency for Science, Technology and Research (A*STAR), 2 Fusionopolis Way, Singapore 138634, Singapore; jerry_heng_from.tp@imre.a-star.edu.sg (J.Z.X.H.); tangytk@imre.a-star.edu.sg (K.Y.T.)

2 Institute of Chemistry, University of the Philippines Diliman, Quezon City 1101, Philippines; mdregulacio@up.edu.ph

* Correspondence: m-lin@imre.a-star.edu.sg (M.L.); lohxj@imre.a-star.edu.sg (X.J.L.); lizb@imre.a-star.edu.sg (Z.L.); yeey@imre.a-star.edu.sg (E.Y.)

† These authors contributed equally.

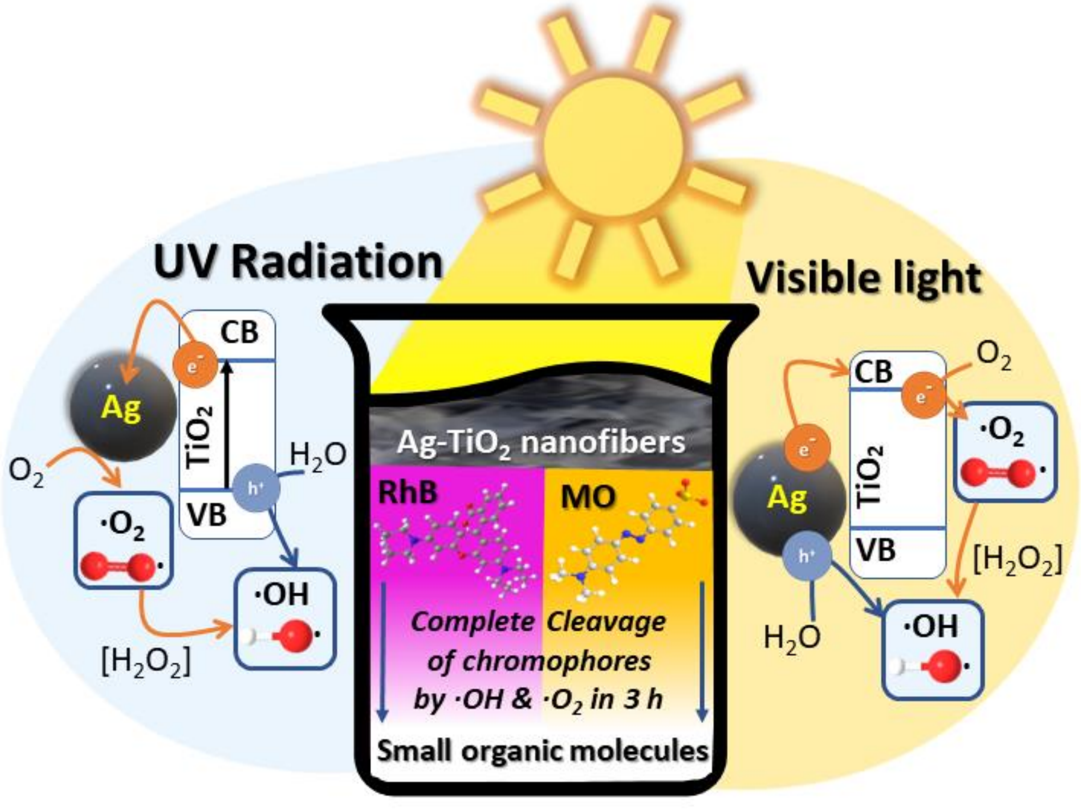

Figure S1. Schematic overview of the solar powered photocatalytic degradation of RhB and MO dyes with Ag–TiO_2 nanofibers as the photocatalyst.

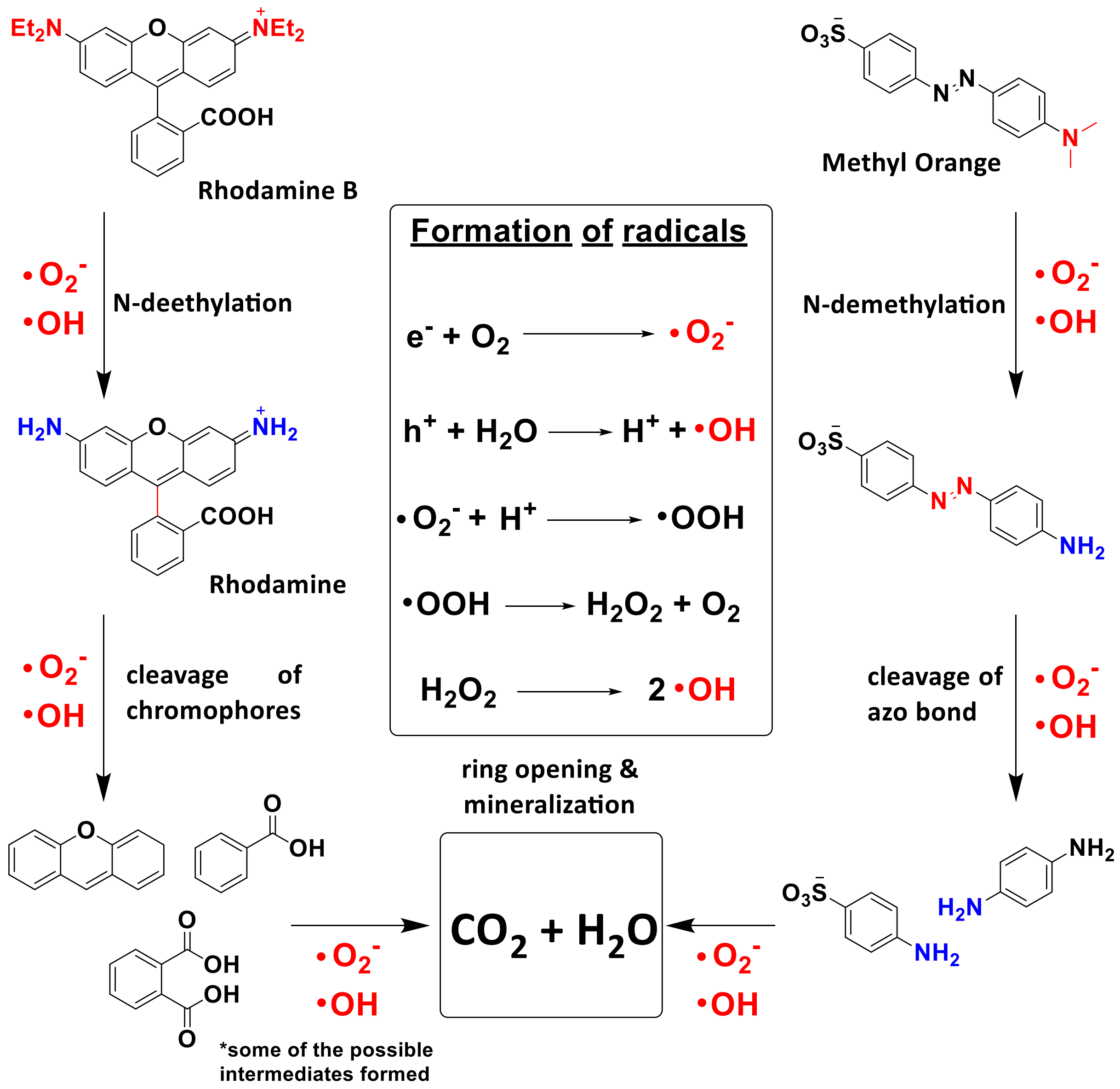

Figure S2. Proposed photodegradation pathway for RhB and MO dyes by $\cdot OH$ and $\cdot O_2^-$ radicals that were generated by electron–hole pairs in Ag–TiO_2 nanofibers.
